# Rare Case Report of Pulmonary Mucosa‐Associated Lymphoid Tissue Lymphoma

**DOI:** 10.1002/rcr2.70284

**Published:** 2025-08-26

**Authors:** Suzhen Yang, Qin Shen

**Affiliations:** ^1^ Department of Respiratory and Critical Care Medicine Hunan Provincial People’s Hospital (The First Affiliated Hospital of Hunan Normal University) Changsha People's Republic of China

**Keywords:** bronchial, lung, mucosa‐associated lymphoid tissue

## Abstract

Bronchial mucosa‐associated lymphoid tissue is the site of the uncommon malignancy known as mucosa‐associated lymphoid tissue (MALT) lymphoma. Due to its lack of distinct clinical signs and imaging characteristics, it is frequently misdiagnosed and underdiagnosed. After receiving unsuccessful treatment for a lung infection or tuberculosis in multiple tertiary care hospitals, we report a male patient who had a solid lesion in his left upper lung for 18 years. The patient was ultimately diagnosed with pulmonary MALT lymphoma at our hospital through a CT‐guided percutaneous lung biopsy using the Jiang technique (i.e., employing a laser‐assisted guidance system in addition to the conventional CT guidance). It is vital to extend one's perspective, synthesise extrapulmonary signs, and work toward the conditions essential to get a high‐quality biopsy specimen when an intrapulmonary lesion is hard to explain by general lung illnesses.

## Graphical abstract

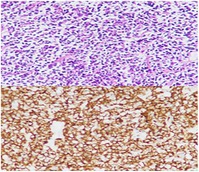

## 1 Introduction

MALT lymphoma is 0.5%–1% of all primary lung cancers [1], which is uncommon. Furthermore, because lung MALT lymphoma is indolent, it is prone to missed and incorrect diagnoses because it lacks characteristic clinical symptoms and imaging findings [2].

## 2 Case Report

**FIGURE 1:**
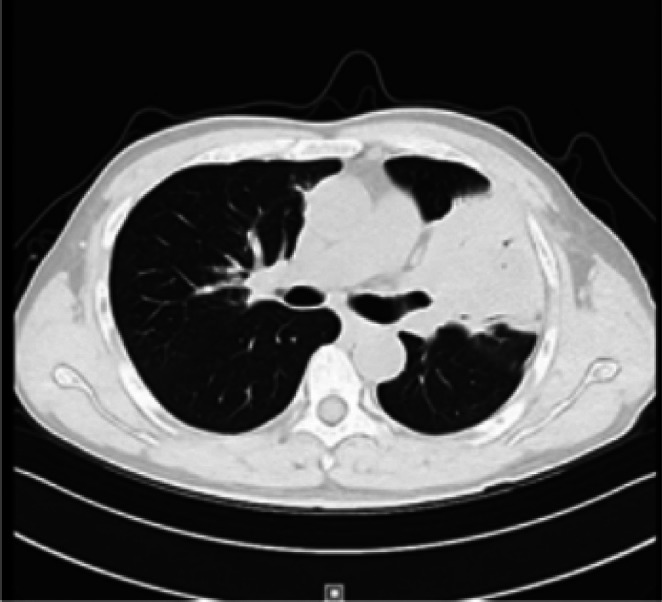
Chest CT suggests solid lesion in left upper lung.

**FIGURE 2:**
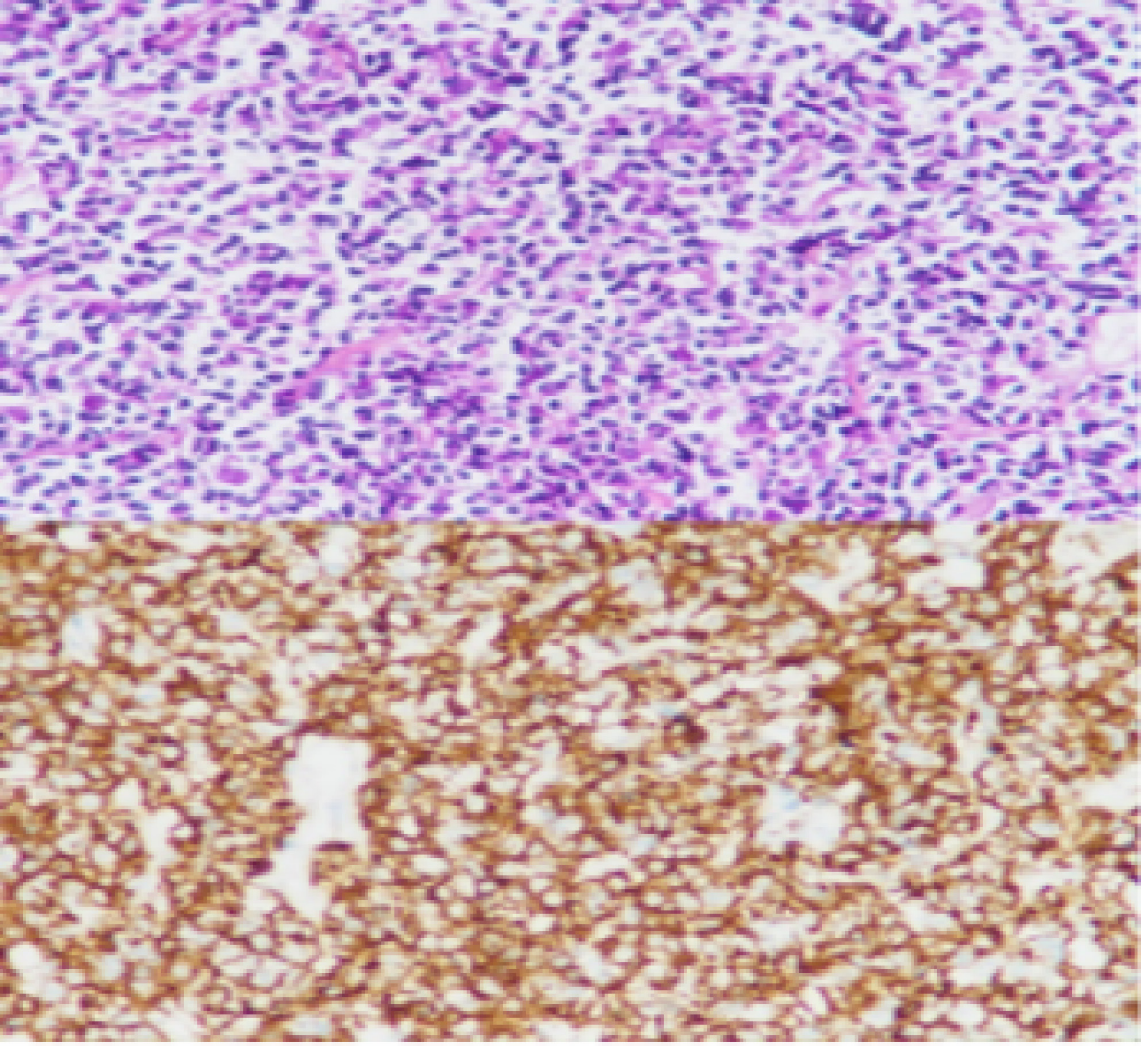
Microscopically, large numbers of lymphocytes were seen infiltrating the surrounding alveoli and interstitium, and proliferative lesions of lymphoid tissue were seen. Immunohistochemistry: CD3(+, T cells), CD20(+), CD21 lamellipodia(+), Ki67(+,20%), CD30(−), CD5(+, T cells), CyclinD1(−), SOX‐11(−), CD10(−), Bcl‐6 scattered(weakly +), Bc1‐2(+), MUM1(+), C‐myc(+0.40%), TdT(−), CD79a(+), CD23(−), CK(pan) tiny foci(+).

**FIGURE 3:**
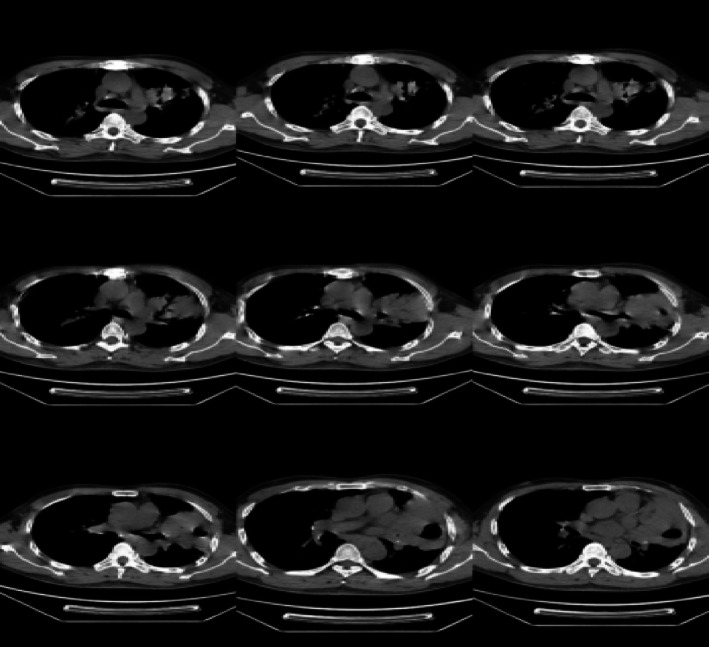
Positron emission tomography‐computed tomography.

**FIGURE 4:**
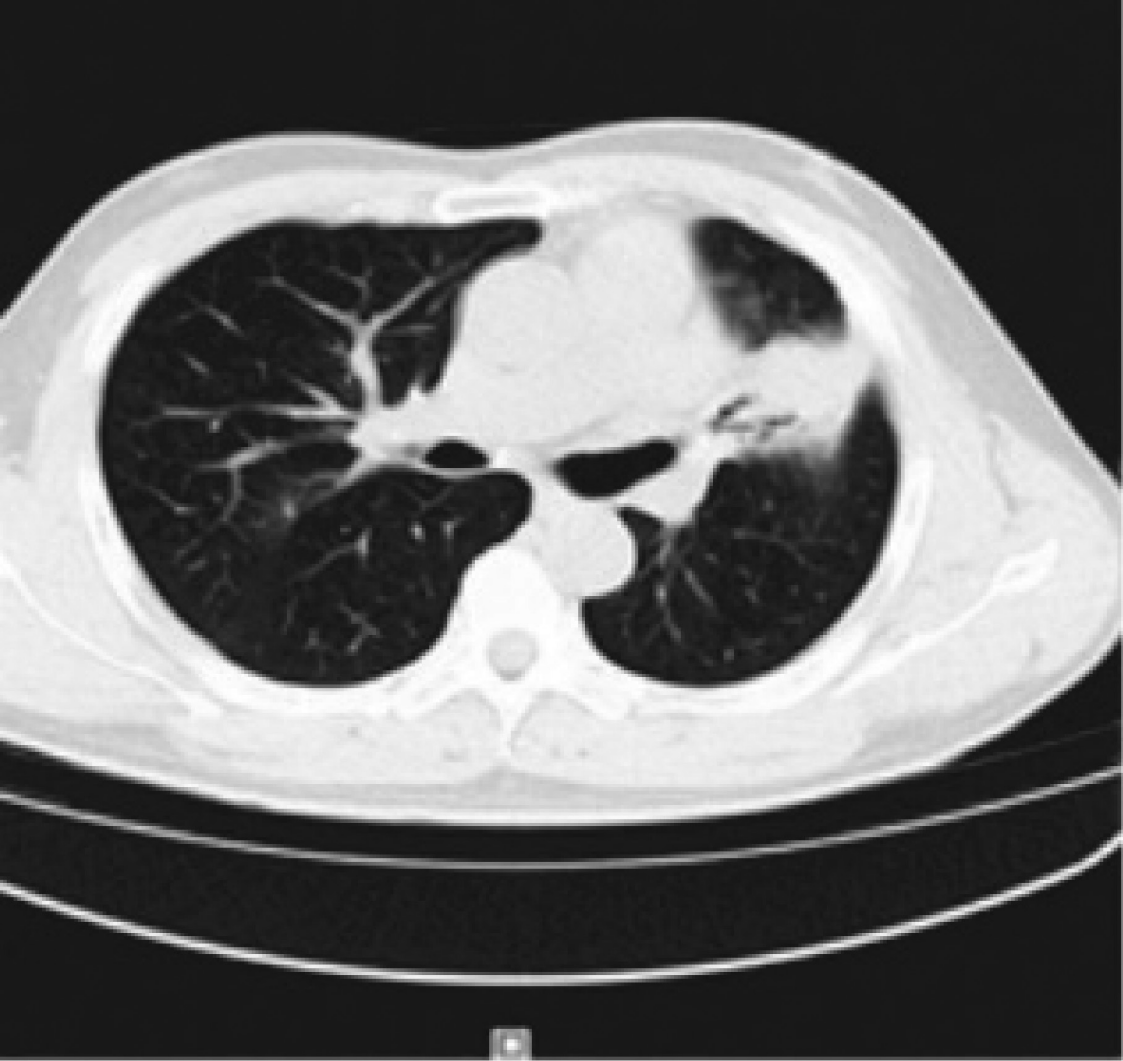
Chest CT after treatment.

A 69‐year‐old male patient presented to our department with recurrent chest tightness, dyspnea, and chest pain for 18 years. Since January 2005, the patient had experienced chest tightness and pain, as well as dyspnea on exertion, without any obvious cause. He had a history of hepatitis B and rheumatoid arthritis, but had not received regular treatment for these conditions. In April 2005, a pulmonary Computed Tomography (CT) scan revealed lesions in the left lingular and right upper lobes, with a high suspicion of secondary pulmonary tuberculosis. The initial CT‐guided percutaneous lung biopsy showed inflammatory changes, and antituberculous treatment (2 months of isoniazid, rifampin, pyrazinamide, and ethambutol followed by 9 months of isoniazid, rifampin, and pyrazinamide was administered) was administered. However, the treatment was not effective. In March 2008, a follow‐up CT scan showed progression of the lesions. Bronchoscopy revealed only nonspecific inflammatory changes. A second percutaneous lung biopsy showed ‘lymphocytic infiltration with focal proliferation,’ suggesting chronic inflammation. Antibiotic treatment was given, but the response was still not satisfactory. From 2016 to 2023, the patient's symptoms of chest tightness and pain gradually worsened. Repeated CT scans of the chest showed consolidation in the left upper lobe with left lung atelectasis, scattered lesions in both lungs, and pleural effusion on the left side. Multiple bronchoscopies during this period also showed bronchial inflammation. Antibiotic treatment was administered, but the response remained poor. No thoracic surgical consultation was obtained from 2005 to 2023 since repeated biopsy results showed no evidence of malignancy. Hepatitis B virus DNA quantification, coagulation function, lupus full set, immunity complete set, liver and kidney function, pathogenicity test, and calcitonin gene were all evaluated and found to be normal. Hepatitis B virus surface antigen was positive, and C‐reactive protein was 30.10 (mg/L). Chest augmentation; rheumatoid factor 321 (IU/ml), CT revealed several enlarged lymph nodes in the cardiogenic angle and mediastinum, as well as massive solid lesions in the left lung (Figure 1). The postoperative lung histology from the CT‐guided percutaneous lung aspiration biopsy was consistent with non‐Hodgkin's B‐cell lymphoma. Mucosa‐associated lymphoid tissue (MALT) lymphoma in the extranodal marginal zone was suggested by immunohistochemistry. EBER(−) for in situ hybridization. CD3(+, T cells), CD20(+), CD21 lamellipodia(+), Ki67(+, 20%), CD30(−), CD5(+, T cells), CyclinD1(−), SOX‐11(−), CD10(−), Bcl‐6 scattered(weakly +), Bc1‐2(+), MUM1(+), C‐myc(+0.40%), TdT(−), CD79a(+), CD23(−), CK(pan) tiny foci(+) immunohistochemistry (Figure 2). Additional PET/CT refinement revealed alterations that were in line with MALT lymphoma infiltration. The patient was diagnosed with MALT lymphoma after taking into account all of the evidence mentioned above (Figure 3). After a comprehensive evaluation of the aforementioned evidence, the patient was diagnosed with MALT lymphoma. Following the diagnosis, the patient was referred to the haematology department for treatment with the BR regimen, which consisted of: rituximab 600 mg in combination with bendamustine 100 mg on Day 1, followed by bendamustine 100 mg alone on Day 2, with each cycle lasting 28 days and a total of four cycles administered. Subsequently, the patient received four cycles of maintenance therapy with rituximab 600 mg. After one cycle of treatment, the patient's clinical symptoms and imaging findings showed significant improvement (Figure 4). During treatment, only Grade 1 fatigue (not affecting daily activities) was observed according to CTCAE v5.0 criteria, with the chest pain VAS score decreasing from 4 at baseline to 2 post‐treatment. At the 6‐month follow‐up after chemotherapy completion, the patient no longer experienced any discomfort symptoms including chest tightness, shortness of breath, or chest pain. No ≥ Grade 2 haematological or non‐haematological toxicities occurred throughout the entire treatment course.

## 3 Discussion

Lung MALT lymphoma is an uncommon kind of non‐Hodgkin's lymphoma that develops from lymphoid tissues linked with the bronchial mucosa. The cause of lung MALT lymphoma is still unknown, and the potential causes of our patient's frequent misdiagnosis and omission of the disease could be Chronic antigenic stimulation brought on by infections or autoimmune diseases may be the secondary aetiology of MALT lymphoma [3]. Therefore, we hypothesise that the patient may have had an inflammatory lesion in the early stages of the disease, but that the disease eventually developed as a result of chronic antigenic stimulation for up to 18 years because the patient's autoimmune disease was not adequately treated or the infected lesions in the lungs were not fully cleared. In the early stages, patients with pulmonary MALT lymphoma typically exhibit nonspecific recurring respiratory infections, including cough, dyspnea, malaise, and chest discomfort, rather than typical or distinctive clinical signs [2]. Due to this characteristic, the patient was often misdiagnosed as having both tuberculosis and a lung infection. Because pulmonary MALT lymphoma tumour cells adhere well, sputum exfoliative cell examination is generally negative; additionally, the majority of the disease's lesions are found in the bronchial tubes beneath the segments, and the tumour cells proliferate infiltratively in the bronchial tubes' submucosa, so a bronchoscopic naked‐eye examination is typically anticipated [4, 5]. As a result, this makes detecting pulmonary MALT lymph much more challenging. The diagnosis of pulmonary MALT lymphoma primarily relies on pathological tissue biopsy and immunohistochemical examination [5]. Although the patient had undergone multiple CT‐guided percutaneous lung biopsies at other hospitals without tumour detection, several possibilities may explain this: First, the positive yield of CT‐guided biopsies depends on various factors, including operator expertise and inaccurate targeting of the puncture site. The successful biopsy at our institution can be attributed to the integration of a laser‐assisted navigation system with conventional CT guidance, which significantly improves targeting accuracy, thereby markedly increasing the procedural success rate. Second, the minimal tissue obtained from previous minimally invasive biopsies might have been insufficient to reliably distinguish between reactive hyperplasia and neoplastic proliferation [6]. Currently, there is no unified global standard treatment protocol for pulmonary MALT lymphoma. For pulmonary MALT lymphoma associated with chronic infections (such as *Helicobacter pylori* or hepatitis C virus infection), eradicating the source of infection may be effective. Treatment strategies need to be individualised based on factors such as the patient's clinical stage, extent of lesions, symptoms, and physical condition. Early‐stage patients may opt for surgery or radiotherapy. For late‐stage patients without significant symptoms or with slow disease progression, a ‘watch and wait’ strategy may be chosen to avoid unnecessary treatment side effects, with intervention initiated when disease progression or symptoms occur [7]. The pathological features of pulmonary MALT lymphoma are relatively complex. The morphology of the tumour cells may be similar to that of reactive lymphoid hyperplasia, and the interpretation of immunohistochemical markers also presents certain difficulties. These factors may lead to delayed diagnosis or misdiagnosis in pathological evaluations.

## Author Contributions

**Suzhen Yang:** responsible for the clinical diagnosis and treatment follow‐up of patients, collecting and organising their clinical data, including symptoms, examination results, and changes in their condition during the treatment process. At the same time, I led the initial drafting of the case report and made significant contributions to the clinical analysis of the case. **Qin Shen:** with professional knowledge, in‐depth analysis of the pathological and physiological mechanisms related to respiratory diseases in the cases provided a solid theoretical basis for the report. In the process of revising the report, we focused on improving the discussion section to make it more scientific and logical.

## Consent

The authors declare that appropriate written informed consent was obtained for the publication of this manuscript and accompanying images.

## Conflicts of Interest

All authors declare no potential conflicts of interest. No financial support was received from any organization for the submitted work, and no financial relationships with any entities that might have an interest in the submitted work exist within the past three years.

## Data Availability

The data that support the findings of this study are available on request from the corresponding author. The data are not publicly available due to privacy or ethical restrictions.
